# Denervation Causes Fiber Atrophy and Myosin Heavy Chain Co-Expression in Senescent Skeletal Muscle

**DOI:** 10.1371/journal.pone.0029082

**Published:** 2012-01-03

**Authors:** Sharon L. Rowan, Karolina Rygiel, Fennigje M. Purves-Smith, Nathan M. Solbak, Douglas M. Turnbull, Russell T. Hepple

**Affiliations:** 1 Muscle and Aging Laboratory, Faculty of Kinesiology, University of Calgary, Calgary, Canada; 2 Mitochondrial Research Group, Institute for Ageing and Health, University of Newcastle Upon Tyne, Newcastle, United Kingdom; 3 Critical Care Division, Royal Victoria Hospital and Department of Medicine, McGill University, Montreal, Canada; Tokyo Medical and Dental University, Japan

## Abstract

Although denervation has long been implicated in aging muscle, the degree to which it is causes the fiber atrophy seen in aging muscle is unknown. To address this question, we quantified motoneuron soma counts in the lumbar spinal cord using choline acetyl transferase immunhistochemistry and quantified the size of denervated versus innervated muscle fibers in the gastrocnemius muscle using the *in situ* expression of the denervation-specific sodium channel, Nav_1.5_, in young adult (YA) and senescent (SEN) rats. To gain insights into the mechanisms driving myofiber atrophy, we also examined the myofiber expression of the two primary ubiquitin ligases necessary for muscle atrophy (MAFbx, MuRF1). MN soma number in lumbar spinal cord declined 27% between YA (638±34 MNs×mm^−1^) and SEN (469±13 MNs×mm^−1^). Nav_1.5_ positive fibers (1548±70 μm^2^) were 35% smaller than Nav_1.5_ negative fibers (2367±78 μm^2^; P<0.05) in SEN muscle, whereas Nav_1.5_ negative fibers in SEN were only 7% smaller than fibers in YA (2553±33 μm^2^; P<0.05) where no Nav_1.5_ labeling was seen, suggesting denervation is the primary cause of aging myofiber atrophy. Nav_1.5_ positive fibers had higher levels of MAFbx and MuRF1 (P<0.05), consistent with involvement of the proteasome proteolytic pathway in the atrophy of denervated muscle fibers in aging muscle. In summary, our study provides the first quantitative assessment of the contribution of denervation to myofiber atrophy in aging muscle, suggesting it explains the majority of the atrophy we observed. This striking result suggests a renewed focus should be placed on denervation in seeking understanding of the causes of and treatments for aging muscle atrophy.

## Introduction

Aging of skeletal muscle is characterized by a decline in mass and function, a process known as sarcopenia [1]. Although many ideas continue to be explored concerning the causes of sarcopenia, one of the first and most resilient ideas involves denervation of muscle fibers [2], [3], an idea first suggested from a report noting marked alterations in the end plate morphology at the neuromuscular junction with aging [4]. Since that time, several lines of evidence have developed showing that denervation occurs in aging muscles, including a progressive reduction in the number of motoneurons in the spinal cord beginning at ≈60 y [5], loss of motoneurons in the periphery [6], [7], fiber type grouping [8], [9], degeneration of neuromuscular junctions [4], [10], loss of motor units [11], [12], and grouped fiber atrophy in aging muscle [13], [14]. It is also striking that the muscle morphological alterations in aging human muscle, e.g., accumulation of severely atrophic angular fibers [14], [15], [16], are similar to those seen in motoneuron diseases and in experimental models of denervation [17], [18], [19]. Finally, some fibers in aged muscles express markers of denervation, such as neural cell adhesion molecule [20] and the sodium channel, Nav_1.5_ [21]. In regard to Nav_1.5_, previous studies have shown this isoform disappears shortly after birth and remains at undetectable levels in healthy adult muscle [22], [23]. However, Nav_1.5_ reappears following denervation in adult muscle [23] and thus, provides an effective method for identifying denervated myofibers in aged muscles.

Whereas the occurrence of denervation in aged muscles is well established, the contribution of denervation to myofiber atrophy and other well-known changes in aging muscle is unknown. In particular, the role of denervation in the age-related shifts in myosin heavy chain (MHC) expression that are the primary basis for fiber type classification, and the frequent MHC co-expression seen in aging muscle [16], [24], [25], changes that are also seen in experimentally denervated muscle [26], has never been quantified in aging muscle. Understanding the quantitative impact of denervation on myofiber atrophy and classical morphological alterations in aging muscle is critical to our understanding of what causes sarcopenia, where current thinking implicates a dizzying multiplicity of factors including impaired muscle protein synthesis [27], enhanced protein degradation [28], elevated myostatin or other TGF-β family members [29], mitochondrial dysfunction [30], and impaired myogenic progenitor cell number and/or function [31], amongst many others.

On the basis of the aforementioned uncertainty about the causes of muscle atrophy with sarcopenia, the primary purpose of this study was to evaluate the hypothesis that denervation causes myofiber atrophy in aging muscle, which is one of the most important phenotypes involved in the reduction of muscle mass and strength with aging. Secondly, based upon the previous suggestion that denervation may also be responsible for the extensive MHC co-expression seen in aging muscles [25], we examined the degree to which denervation co-localizes with MHC co-expression. To meet these objectives, we employed the use of a well-validated model of sarcopenia, the Fisher 344×Brown Norway F1-hybrid (F344BN) rat, and studied an age range associated with a marked degree of muscle atrophy [32], [33], [34]. The value of the F344BN rat model is that this strain has a longer lifespan and exhibits less age-related pathology than classical inbred rat strains like the Fischer 344 rat [35], [36]. Its trajectory of age-related muscle mass decline is similar to that observed in humans when examined at similar relative time-points in the lifespan [34], and key morphological changes associated with denervation-reinnervation seen in humans with advancing age, such as fiber type grouping [8], are also seen in this model [37]. We quantified motoneuron soma numbers in lamina IX of the ventral horn in the lumbar spinal cord, and identified denervated myofibers in relation to myofiber MHC expression in the red region of gastrocnemius (Gas) muscle by examining the myofiber expression patterns of the sodium channel, Nav_1.5_, which is the isoform appearing in adult muscle fibers following denervation [22],[23]. To gain insights into the mechanisms driving atrophy of denervated myofibers, we also examined *in situ* abundance of two proteins necessary to target myofibrillar and other proteins for degradation by the proteasome with various atrophy stimuli, the ubiquitin ligases muscle atrophy f-box (MAFbx) and muscle ring finger-1 (MuRF1) [38].

## Results

### Spinal Cord Motoneuron Counts

**Figure 1:**
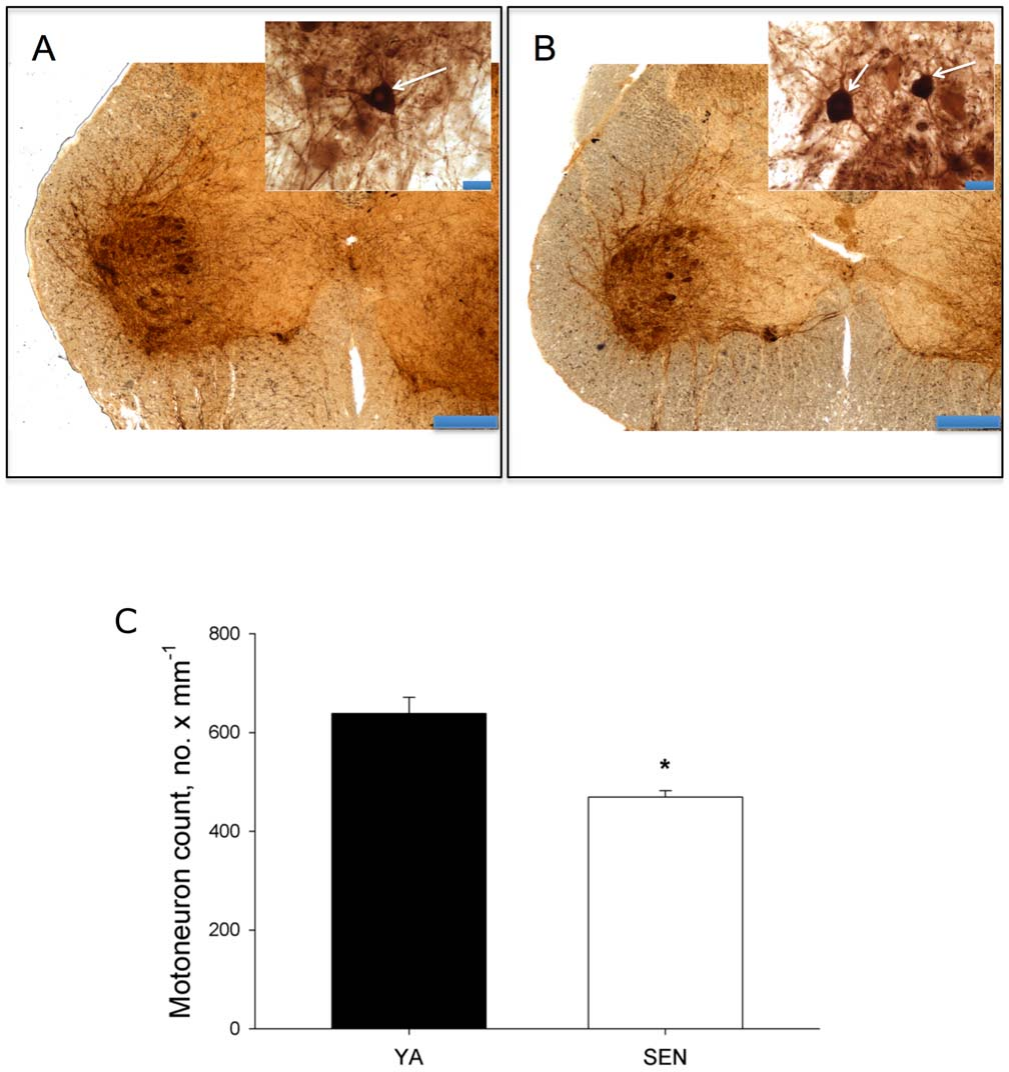
Spinal cord motoneuron counts. A: Photomicrograph of choline acetyl transferase labeled cross-sections of the spinal cord from a young adult rat and B: senescent rat (scale bar = 400 µm). Insets for both panels are higher power images showing motoneuron soma (arrows) (scale bar = 40 µm). C: mean motoneuron counts for young adult (YA) and senescent (SEN) rats. *P<0.05 versus YA.

Figure 1 depicts representative photomicrographs of choline acetyltransferase immunohistochemically-labelled cross-sections of the lumbar spinal cord from a young adult (Fig. 1A) and senescent (Fig. 1B) animal. There was a 27% decline in motoneuron soma between young adult and senescent animals (Fig. 1C).

### Muscle Mass, Fiber Size and MHC Expression

**Figure 2:**
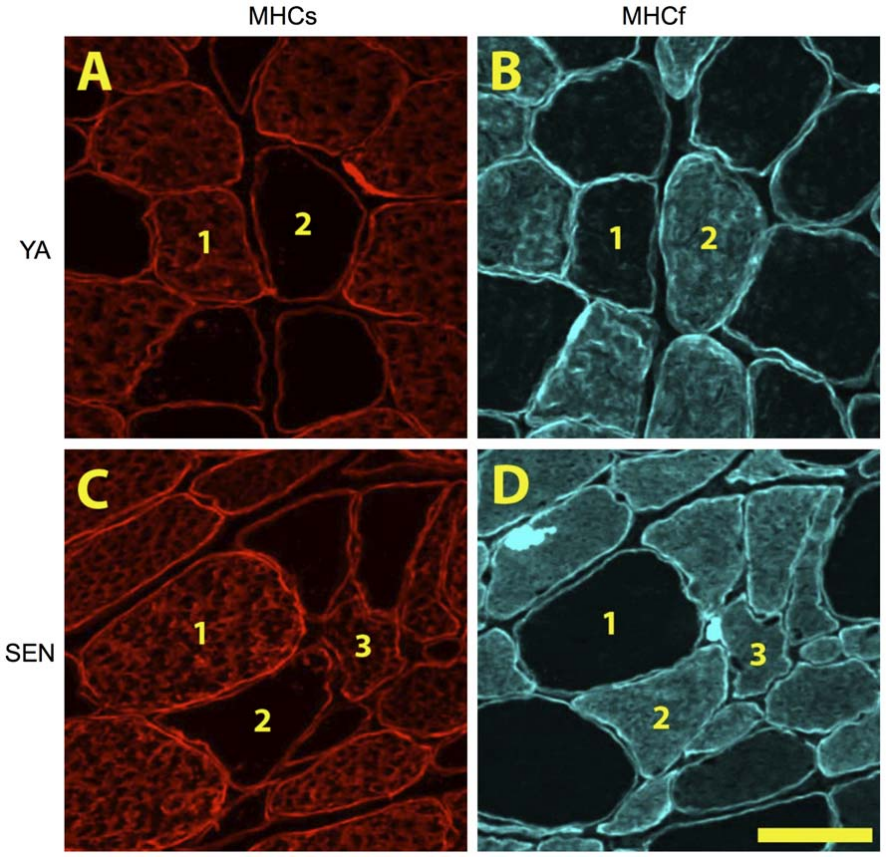
Fiber type changes with aging. Photomicrographs of serial sections labeled for myosin heavy chain (MHC) slow (A,C) and MHC fast (B,D) within the red region of gastrocnemius muscle from a young adult (A,B) and senescent (C,D) rat. In these images 1 denotes a MHCs fiber, 2 denotes a MHCf fiber, and 3 denotes a fiber co-expressing both MHCs and MHCf. Scale bar is 50 µm.

**Figure 3:**
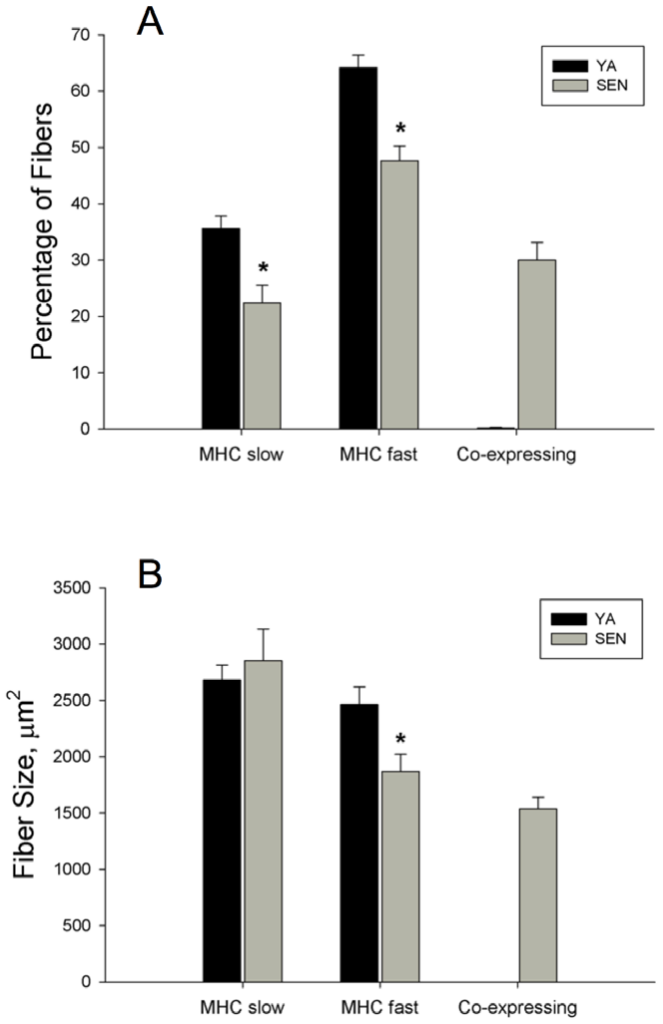
Fiber type proportion and fiber size in young adult (YA) and senescent (SEN) muscle. Panel A depicts the percentage of fibers in each MHC class examined (*P<0.05 vs YA; too few MHC co-expressing fibers in YA to permit statistical comparison to SEN). Panel B depicts differences in fiber size according to each MHC class examined (*P<0.05 vs YA).

Gas muscle mass was reduced by 38% from (2054±41 mg) to SEN (1277±36 mg; P<0.05). Figure 2 shows representative photomicrographs of cross-sections of the Gas muscle immunolabelled for dystrophin and MHC slow or MHC fast, with examples of pure MHC slow, pure MHC fast, and MHC co-expressing fibers in SEN muscle. Based upon measures made in an average of 96±5 fibers per muscle, the proportion of MHC slow fibers decreased 39% from YA (36±2%) to SEN (22±3%; P<0.05) (Fig. 3A). Similarly, the proportion of MHC fast fibers decreased 25% from YA (64±2%) to SEN (48±3%; P<0.05). MHC co-expression was seen in only two fibers from one YA muscle, but this trait was very common in all of the SEN muscles, with an average of 30±3% of all fibers within the red region of Gas muscle exhibiting MHC co-expression. The fact that the abundance of both MHC slow and MHC fast fibers decreased with aging suggests the origin of the MHC co-expressing fibers was from both formerly pure MHC slow and formerly pure MHC fast fibers.

Mean fiber area of MHC slow fibers was not different between YA (2683±132 µm^2^) and SEN (2851±282 µm^2^) (Fig. 3B). In contrast, the mean fiber area of MHC fast fibers decreased by 24% in SEN (1869±153 µm^2^) versus YA (2464±156 µm^2^; P<0.05). The mean fiber area of MHC co-expressing fibers in SEN was 1538±102 µm^2^ and was significantly smaller than both MHC slow (46%) and MHC fast fibers (18%) in SEN (P<0.05).

### Myofiber Nav_1.5_ Expression

**Figure 4:**
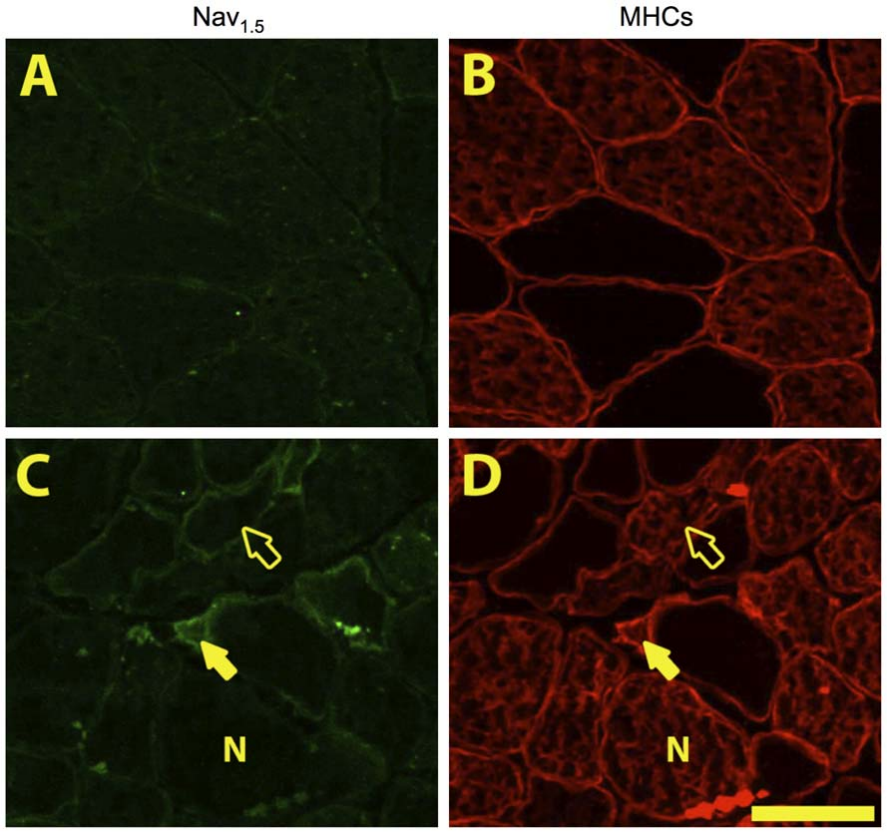
Alterations in myofiber Nav_1.5_ expression with aging. Photomicrographs of serial sections of the red region of gastrocnemius muscle labeled for Nav_1.5_ (A,C) and MHC slow (B,D) in young adult (A,B) and senescent (C,D) rats. In senescent muscle we saw three classes of Nav_1.5_ labelling; negative (N), ringed (displaying circumferential labelling, open arrowhead) and cytoplasm (showing cytoplasmic staining, shaded arrowhead). Scale bar is 50 µm.

Nav_1.5_ negative controls (no incubation with primary antibody) and a Nav_1.5_ positive control (rat heart tissue) (see Figs. S1 and S2) demonstrated minimal background labelling in the absence of the primary antibody, and the expected high Nav_1.5_ expression levels in heart where it is the dominant isoform expressed [39]. No Nav_1.5_ positive fibers were seen in YA muscle (Fig. 4A), but in SEN muscles we observed two distinct types of Nav_1.5_ labeling (Fig. 4C), one characterized by circumferential ringing (ringed fibers), and another characterized by cytoplasmic labelling (cytoplasm fibers).

**Figure 5:**
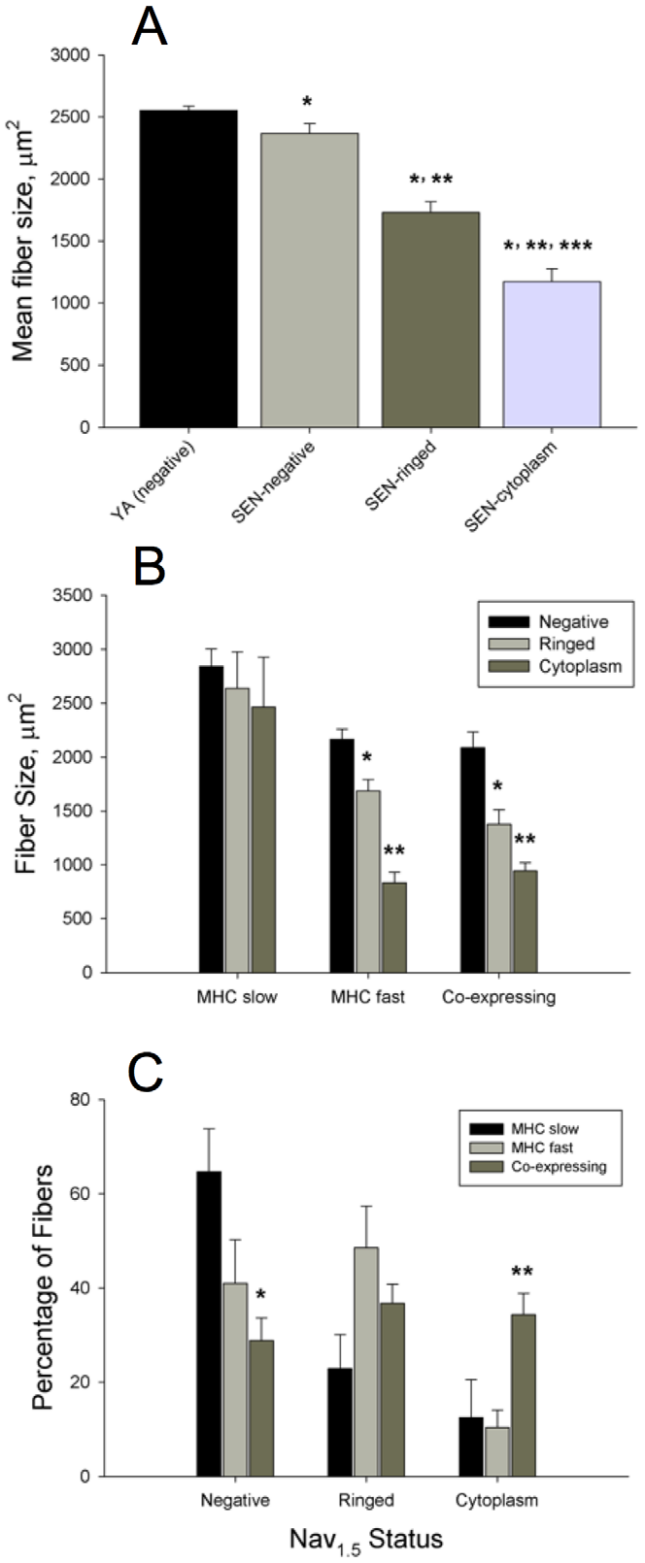
Fiber size in denervated versus innervated myofibers. A: the mean fiber size of young adult (YA) myofibers where no Nav_1.5_ labeling was seen, versus Nav_1.5_ negative senescent (SEN) myofibers, Nav_1.5_ positive SEN ringed fibers, and Nav_1.5_ positive SEN cytoplasm fibers (*P<0.05 vs YA, **P<0.05 vs SEN negative, ***P<0.05 vs SEN ringed). B: the size of Nav_1.5_ positive fibers versus negative fibers according to the MHC labeling category in SEN muscle (*P<0.05 vs Nav_1.5_ negative, **P<0.05 vs ringed). C: the proportion of fibers within a given MHC labeling category that are Nav_1.5_ negative, Nav_1.5_ positive ringed, and Nav_1.5_ positive cytoplasm in SEN muscle (*P<0.05 vs MHC slow, **P<0.05 vs MHC slow and MHC fast).

In SEN animals, the mean fiber area of Nav_1.5_ negative, Nav_1.5_ ringed and Nav_1.5_ cytoplasm positive fibers was determined (Figure 5A), based upon measures made in an average of 94±12 fibers per muscle. This analysis revealed that Nav_1.5_ ringed fibers (1730±88 μm^2^) were 27% smaller than Nav_1.5_ negative fibers (2367±78 μm^2^; P<0.05). Similarly, Nav_1.5_ cytoplasm positive fibers (1172±104 μm^2^) were 32% smaller than Nav_1.5_ ringed fibers (P<0.05) and 51% smaller than Nav_1.5_ negative fibers (P<0.05). Strikingly, the mean fiber size of Nav_1.5_ negative fibers in SEN (2367±78 μm^2^) was only 7% smaller than the mean fiber size in YA (2553±33 μm^2^; P<0.05) where no Nav_1.5_ positive fibers were identified.

In MHC slow fibers, the mean size of Nav_1.5_ negative (2842±160 µm^2^), ringed (2638±339 µm^2^), and cytoplasm (2464±465 µm^2^) fibers was not different (Fig. 5B). Furthermore, there was no difference in the size of MHC slow fibers between YA (2686±128 µm^2^) and SEN regardless of the Nav_1.5_ labeling status. Although this might be taken to indicate that denervated MHC slow fibers do not atrophy, it is noteworthy that 75% of the MHC slow fibers ≤1000 μm^2^ in size were positive for Nav_1.5_. In contrast to MHC slow fibers, Nav_1.5_ ringed MHC fast fibers (1687±104 µm^2^) were 22% smaller than Nav_1.5_ negative fibers (2165±94 µm^2^; P<0.05). Additionally, MHC fast Nav_1.5_ cytoplasm fibers (835±98 µm^2^) were 61% smaller than Nav_1.5_ negative fibers (P<0.05) and 51% smaller than Nav_1.5_ ringed fibers (P<0.05). In MHC co-expressing fibers, Nav_1.5_ ringed fibers (1378±133 µm^2^) were 34% smaller than Nav_1.5_ negative fibers (2087±146 µm^2^; P<0.05). Additionally, within MHC co-expressing fibers, Nav_1.5_ cytoplasm fibers (945±76 µm^2^) were 55% smaller than Nav_1.5_ negative fibers (P<0.05), and 31% smaller than Nav_1.5_ ringed fibers (P<0.05).

The percentage of MHC slow, MHC fast and MHC co-expressors exhibiting each of the Nav_1.5_ labelling patterns in SEN muscles is shown in Figure 5C. There was a strong trend for a difference between the proportion of MHC slow (60±11%), MHC fast (45±7%), and MHC co-expressing (29±4%) fibers (P = 0.052) that were Nav_1.5_ negative. There was no difference between the proportion of MHC slow (26±6%), MHC fast (43±6%), and MHC co-expressing (30±4%) fibers that were Nav_1.5_ ringed. Amongst fibers that were Nav_1.5_ cytoplasm positive, there were significantly more MHC co-expressing (41±6%) than MHC slow (14±10%; P = 0.009) or MHC fast fibers (12±3%; P = 0.006). More than 70% of MHC co-expressing fibers exhibited some degree of Nav_1.5_ positive labelling (ringed plus cytoplasm together), suggesting that denervation is the major cause of MHC co-expression in SEN muscles.

The fiber size distribution of Nav_1.5_ negative fibers in SEN (Figure S3A) was normally distributed and is centred on the mean fiber size of YA fibers (YA data not shown). Only 6% of Nav_1.5_ negative fibers have a size ≤1000 µm^2^, a size that is commonly associated with prolonged (2 months) denervation of muscle fibers [40]. In contrast, the fiber size distribution of the Nav_1.5_ ringed fibers appears bimodal, with 39% of these being ≤1000 µm^2^. Interestingly, 90% of the fibers ≤1000 µm^2^ in size were positive for Nav_1.5_, with roughly equal proportions of these coming from the ringed versus cytoplasm labelling categories, illustrating the large contribution of denervation to accumulation of severely atrophied fibers in SEN muscle. Finally, whereas fiber size distributions of Nav_1.5_ ringed or cytoplasm fibers within each MHC classification (Fig. S3B) show very few MHC slow fibers ≤1000 µm^2^, both MHC fast and MHC co-expressors demonstrated a large abundance of fibers ≤1000 µm^2^ in size.

### Myofiber Ubiquitin Ligase Expression

**Figure 6:**
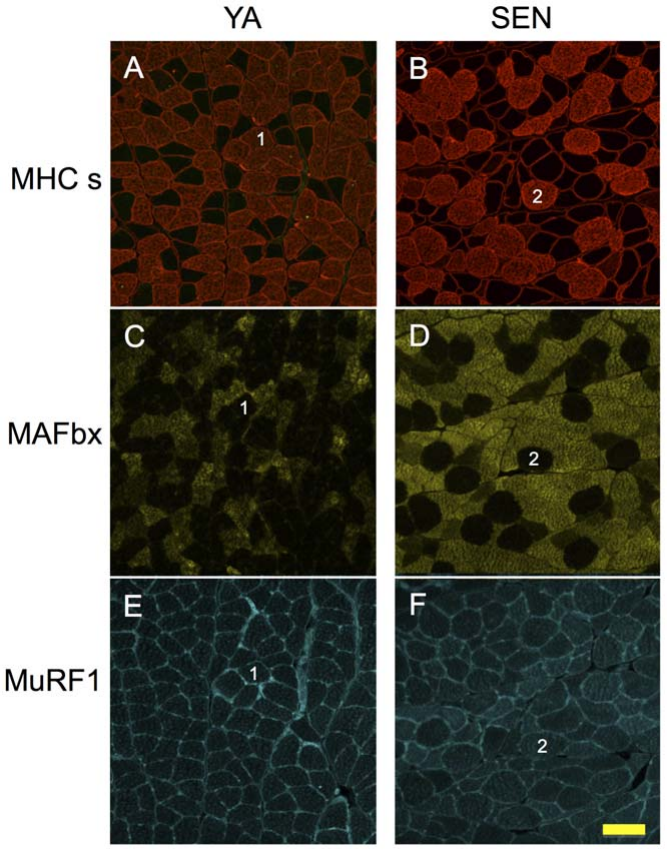
In Situ myofiber ubiquitin ligase expression with aging. Serial images of the red region of gastrocnemius muscle labeled for MHC slow (A,B), MAFbx (C,D) and MuRF1 (E,F) in young adult (YA) (A,C,E) and senescent (SEN) (B,D,F) rats. Fibers sharing the same number are the same fiber in serial sections. Scale bar is 100 µm.

**Figure 7:**
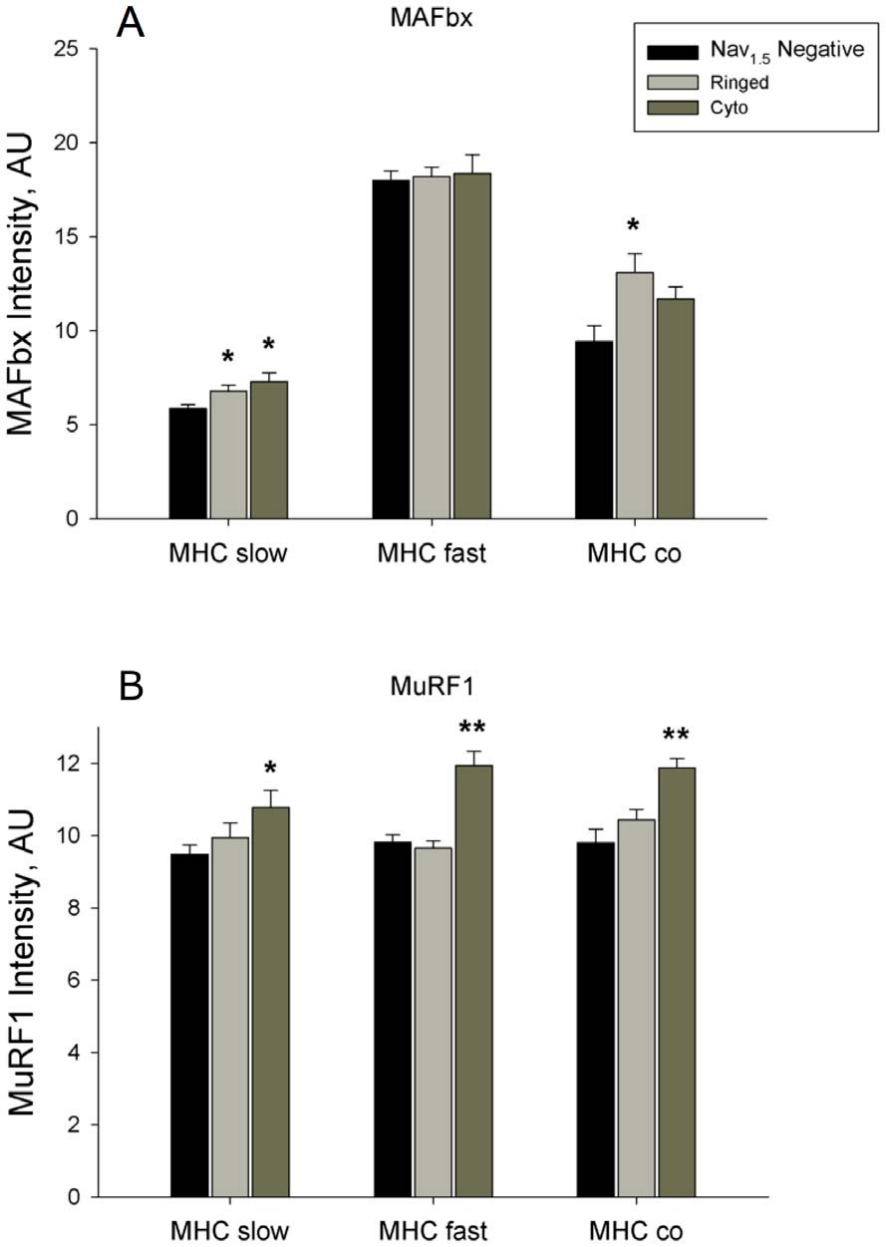
Mean alterations in myofiber ubiquitin ligase expression with aging. *In situ* expression levels of the ubiquitin ligases, MAFbx (A) and MuRF1 (B), in the red region of senescent gastrocnemius muscle in fibers that are MHC slow, MHC fast, and MHC fast/slow co-expressors, in relation to their Nav_1.5_ labeling status (*P<0.05 versus Nav_1.5_ negative, **P<0.05 versus Nav_1.5_ negative and versus Nav_1.5_ ringed).

MAFbx and MuRF1 antibody negative controls (no incubation with primary antibody) demonstrated minimal background labelling in the absence of the primary antibody (see Fig. S4). Figure 6 shows serial sections labeled for MHC slow (Fig. 6A, 6B), MAFbx (Fig. 6C, 6D), and MuRF1 (Fig. 6E, 6F) in YA and SEN animals. MAFbx was elevated in Nav_1.5_ ringed and cytoplasm positive MHC slow fibers (Fig. 7A; P<0.05), whereas MuRF1 was elevated only in Nav_1.5_ cytoplasm positive fibers (Fig. 7B; P<0.05). Conversely, MuRF1 (P<0.05) but not MAFbx was elevated in Nav_1.5_ cytoplasm positive MHC fast fibers. In MHC co-expressing fibers, MAFbx was elevated only in Nav_1.5_ ringed fibers (P<0.05), and MuRF1 was elevated only in Nav_1.5_ cytoplasm positive fibers (P<0.05). MAFbx expression in Nav_1.5_ negative MHC fast fibers was more than 3-fold that of Nav_1.5_ negative MHC slow fibers (P<0.05).

## Discussion

The purpose of this study was to test the hypothesis that denervation causes myofiber atrophy and MHC co-expression in aging muscle by making quantitative measures of the size and MHC expression characteristics of denervated versus innervated myofibers in aging muscle. Consistent with our hypothesis, there was a significant reduction in number of motoneuron soma in the lumbar region of the spinal cord with aging and we show for the first time that fibers expressing the Nav_1.5_ isoform characteristic of denervated myofibers in adult muscle [22], [23] were significantly smaller than Nav_1.5_ negative fibers (presumed to be innervated) in aging muscle. Strikingly, 90% of fibers ≤1000 µm^2^, a size previously shown to accumulate in long-term denervated rat muscle (2 months) [40] and which demarcate the accelerating phase of sarcopenia in both slow and fast muscles [41], were Nav_1.5_ positive, consistent with denervation being the primary cause of their accumulation. Since Nav_1.5_ negative fibers in aging muscle were <7% smaller than fibers in YA muscle, where no Nav_1.5_ positive fibers were seen, these results implicate denervation as the major cause of myofiber atrophy in aging muscle. In addition, this study showed that MHC co-expressing fibers are largely Nav_1.5_ positive in aging muscle, suggesting that denervation is also the primary cause of MHC co-expression in aging muscle. The ubiquitin ligases, MAFbx and MuRF1, demonstrated distinct expression patterns not only between MHC classes but also between Nav_1.5_ positive versus negative fibers, in a manner consistent with a role for the proteasome pathway in atrophy of denervated fibers in aging muscle. Collectively, these results provide the first quantitative data consistent with denervation being a primary cause of MHC co-expression and myofiber atrophy at the single myocyte level in aging muscles, and show that the atrophy occurs, in part, through activation of the proteasome proteolytic machinery.

### Motoneuron Loss from the Spinal Cord with Aging

A significant decline in spinal cord motoneurons with aging was previously shown in humans [5] and has also been shown in some animal models of aging [42], [43]. Similarly, previous studies in both humans [11], [12] and animal models [7], [9], [44] are consistent with a loss of motor units with aging. In contrast, a recent study examining this issue in the F344BN rat (the same model used here) reported no significant change in motoneuron numbers in the lumbar spinal cord of 31 mo old female rats [45]. This contrasts with the current results using the same animal model showing a significant decline in motoneuron number in the lumbar spinal cord of 36 mo old male F344BN rats. Key differences in our study are the more advanced age of the animals and the use of male animals. Interestingly, we have previously observed that female F344BN rats even at 33 mo of age have lost only 13% of Gas muscle mass whereas male F344BN rats at 33 mo of age have lost about 30% of Gas muscle mass (R.T. Hepple, unpublished observations). As such, the preservation of motoneuron number reported in female rats previously [45] is consistent with their relative resistance to aging muscle atrophy. It is also important to note that we do not expect a directly proportional relationship between the fraction of denervated fibers and degree of motoneuron loss, in part because some denervation may arise through myocyte-driven pathways [46], [47] and because the number of fibers per motor unit increases with aging [9], [48]. Both of these effects would mean that the proportion of denervated fibers in a given muscle could exceed the loss of motoneurons. In addition, since the degree of atrophy between muscles can vary considerably with aging [32], the susceptibility to denervation may also be expected to vary between muscles. Finally, it is also important to note that a depletion of gastrocnemius motoneurons with aging in the rat has been previously established [7]. Thus, although the motoneuron counts in the lumbar spinal are not solely the reflection of motoneuron loss in the gastrocnemius muscle, they provide a general index of the degree of motoneuron depletion with aging in the hindlimb.

### Denervation in Aging Muscle and Age-related Changes in Myofiber Size

A decline in myofiber size is a well-know change in aging muscles [13], consistent with the current results. Interestingly, although denervation clearly occurs in aging muscle [9], [21], [49], and it is well established that experimental models of denervation cause muscle atrophy [40], [50], there are no quantitative data documenting the degree to which denervation causes the well-known myofiber atrophy with aging.

To address the quantitative role of denervation in aging myofiber atrophy, this study examined the expression of the sodium channel isoform, Nav_1.5_, to identify denervated myofibers. In skeletal muscle, Nav_1.5_ is the dominant isoform during embryonic myogenesis and it is gradually replaced by the adult isoform, Nav_1.4_, following establishment of innervation and muscle activity [22]. Nav_1.5_ re-appears in adult skeletal muscle following denervation [22], [23], appearing first at end plate regions, followed by extrajunctional expression with longer duration of denervation [51], and declining as innervation is re-established [52]. Previous studies have established that expression of Nav_1.5_ alters the electrophysiological properties of the myocyte, including a resistance to tetrodotoxin treatment [21], [22], but its role in promoting reinnervation of denervated myofibers or other physiologically relevant functions, remains unclear. Interestingly, the current results show that Nav_1.5_ positive fibers exhibit marked atrophy compared to Nav_1.5_ negative fibers in SEN muscles, with 39% of Nav_1.5_ positive fibers (ringed and cytoplasm combined) having a size of ≤1000 μm^2^. This is a size typical of long-term (2 month) denervated rat muscle fibers [40] and a size that tracks the trajectory of sarcopenia in fast and slow muscle [41]. Furthermore, 90% of the fibers ≤1000 μm^2^ were positive for Nav_1.5_ expression, consistent with denervation being the primary cause of the accumulation of these very small muscle fibers. Although the severity of whole muscle atrophy that we observed (38% less in SEN versus YA) is in excess of that which can be explained by the selective atrophy of denervated fibers in the current study, the disparity can be reconciled by the permanent loss of long-term denervated myofibers [50].

An important caveat to our measures is that since we make our measures in tissue cross-sections, we cannot be certain that the phenotypes observed (e.g., MHC expression, Nav_1.5_ status) are constant along the length of the muscle fiber. However, in other experiments (R.T. Hepple and F.M. Purves-Smith, unpublished) we find that the MHC expression remains constant in the vast majority of fiber profiles in serial sections spanning 24 μm of muscle fiber length. Since myonuclei occur approximately one every 15 μm of fiber length [53], this suggests that the MHC expression pattern (and thus, other phenotypes under nuclear control) are constant between at least two myonuclear domains in the vast majority of aging muscle fibers.

To put this study in the broader context of sarcopenia, based upon survival curves, the age of the animals studied (36 mo of age, approximately 35% survival rate) is similar to an 85 y old human [36], [54], and represents an age where the health consequences of sarcopenia are most severe [55]. In addition, the degree of muscle atrophy observed (38% loss of Gas mass compared to YA animals) indicates an advanced stage of sarcopenia comparable to what is seen in humans beyond the 8^th^ decade of life [55]. As such, these results showing that denervation is likely the primary factor causing myofiber atrophy in muscle at a severe stage of sarcopenia strongly implicates denervation in precipitating the most adverse consequences of sarcopenia in advanced age.

### Myocyte- versus Motoneuron-driven Mechanisms of Denervation

An important issue raised more than 20 years ago was the idea that myocyte-driven mechanisms may contribute to denervation-reinnervation cycles in aging muscles. Specifically, the pioneering work of Balice-Gordon using repeated imaging of the same neuromuscular junctions over the lifespan of mice found marked fragmentation of the neuromuscular junction with advancing age [47]. As these changes in the neuromuscular junction began well before an age where motorneuron loss from the spinal cord has been shown to occur, this argues strongly for events occurring within the muscle rather than spinal cord in causing cycles of denervation of aging myocytes. However, it is important to note here that since the abundance of the severely atrophied fibers characteristic of long term denervation remains at low abundance until very advanced age [37], [56], and fiber loss is slow at early phases of aging muscle atrophy and accelerates in late stages where muscle atrophy also accelerates [57], most fibers that become denervated prior to the accelerating phase of aging muscle atrophy must be reinnervated, a point consistent with the profound remodelling of the motor unit with aging [58].

More recently, elegant studies using transgenic mice affecting different aspects of the agrin-muscle specific kinase (MuSK) signalling axis at the neuromuscular junction showed that agrin deficiency recapitulates key morphological aspects of muscle aging, including neuromuscular junction fragmentation, fiber type grouping, fiber size heterogeneity, an increase in abundance of fibers expressing more than one MHC isoform (MHC co-expression), and fiber loss [59]. Since many of these morphological changes are indicative of repeating cycles of denervation-reinnervation, and these were shown to occur in the absence of a loss of motoneurons in the spinal cord [59], this provides clear proof-of-concept that mycoyte-driven mechanisms could be an important cause of denervation in aging muscles. As such, it may be that cycles of denervation and reinnervation in aging muscle precede loss of motoneurons in the spinal cord and that the latter event occurs only in very advanced stages of aging muscle atrophy, precipitating the more aggressive decline in muscle mass seen at advanced age [13], [37], [57]. Indeed, it seems likely that the repeating cycles of denervation-reinnervation would cause some motor units to become very large and may result in motoneuron burnout in a similar way as is thought to occur in the muscles of post-Polio syndrome patients years after the initial disease insult [60]. Further studies are clearly required to address these issues.

### Impact of Denervation on Atrophy of Slow versus Fast Myofibers

Our results show that Nav_1.5_ positive MHC slow fibers were more resistant to atrophy compared to Nav_1.5_ positive MHC fast or MHC co-expressing fibers, a finding consistent with better protection of slow twitch fiber size with aging [13]. However, it is noteworthy that the proportion of both MHC slow and MHC fast fibers declined in aging muscle, and that, therefore, both of these fiber populations contributed to the dramatic accumulation of MHC co-expressors with aging. Since the Nav_1.5_ positive MHC co-expressing fibers demonstrated marked atrophy with aging, it seems likely that MHC co-expressing fibers which are derived from denervated MHC slow fibers do in fact undergo substantial atrophy. The unimodal nature of the Nav_1.5_ positive MHC co-expressor size distribution (Fig. S3B) suggests all MHC co-expressing fibers behave as a single population in terms of their atrophy susceptibility and is consistent with this view. Finally, 75% of the MHC slow fibers that exhibited a size of ≤1000 μm^2^ were positive for Nav_1.5_, suggesting that even in MHC slow fibers, denervation is the primary cause of myofiber atrophy with aging.

### Myosin Heavy Chain Co-expression in Aging Muscle

Recent studies show the direction of shift in MHC expression (from fast to slow, or slow to fast) with aging depends upon the muscle examined [61], with the shift generally occurring in the direction of the non-dominant MHC isoform [25]. Furthermore, these shifts in MHC expression are largely due to co-expression of multiple MHC isoforms within single fibers [24], [25], [62], rather than an increase in one pure MHC fiber population at the expense of another. Since experimental denervation of skeletal muscle leads to MHC co-expression within individual myofibers [18], the current experiments directly tested the prior suggestion [25] that denervation may be an important cause of MHC co-expression in aging muscles.

To address this issue, we examined the red region of the Gas muscle because of its mixture of MHC slow and MHC fast (type IIa) fibers [63]. Since there were declines in the proportions of both MHC slow and MHC fast with aging, it appears that the MHC co-expressing fibers in this muscle with aging were derived from both formerly pure MHC slow and formerly pure MHC fast fibers. Additionally, >70% of the MHC co-expressing fibers were positive for Nav_1.5_ expression. Thus, the significance of the current results is they directly demonstrate, for the first time, that denervation is likely the primary cause of MHC co-expression in aged muscles, and as such, plays a key role in the shifts in MHC expression seen in advanced stages of sarcopenia.

### Ubiquitin Ligase Expression in Denervated Myofibers

To examine the mechanisms driving atrophy of Nav_1.5_ positive fibers, the current study also quantified *in situ* expression levels of the ubiquitin ligases, MAFbx and MuRF1; proteins that are necessary to induce atrophy under a variety of conditions, including denervation [38], [64], [65]. It was previously shown that both MAFbx and MuRF1 are elevated at the protein level in aged sarcopenic muscle [28], [66]. The current results show that both MAFbx and MuRF1 are particularly elevated in Nav_1.5_ positive fibers in SEN muscle, suggesting that accumulation of denervated fibers is an important factor driving the increase in ubiquitin ligase protein expression in aging muscles. Since the transcriptional response of MAFbx may be more rapid than MuRF1 following denervation or other atrophy stimuli [64], [65], the fact that MuRF1 was elevated only in Nav_1.5_ cytoplasm positive fibers is consistent with these being fibers that have been denervated for a longer time than Nav_1.5_ ringed fibers, and likely also contributes to the greater atrophy seen in Nav_1.5_ cytoplasm fibers.

An important point of consideration is whether the rather modest increases in MAFbx and MuRF1 protein abundance (10–30%) are functionally relevant in explaining the marked atrophy we saw. Although there are multi-fold increases in MAFbx and MuRF1 transcript levels with atrophy stimuli [38], [64], [67], we are not aware of any studies documenting the degree of elevation in the protein levels of MAFbx and MuRF1 necessary to cause atrophy. Further to this, it is possible that post-translational modification (e.g., phosphorylation of specific amino acid residues) of MAFbx and MuRF1 could further modulate the biological activity of these proteins beyond an increase in their expression levels *per se*, particularly in the MHC fast fibers where Nav_1.5_ expression in the sarcolemmal region was not associated with an induction of either MAFbx or MuRF1 despite the significant atrophy observed. On the other hand, these modest changes in ubiquitin ligase levels coupled with an increase in myofibrillar protein substrate subsequent to elevated levels of calpains following denervation [68] might be sufficient to induce greater protein degradation independent of changes in the atrophy-inducing ubiquitin ligases *per se*. Consistent with this latter view, the slow time-course of denervation atrophy, where it takes approximately two months for myofibers to atrophy from values typical of innervated fibers to a size of ≤1000 μm^2^ [40], implies that only small increases in protein degradation are involved in denervation atrophy. These issues are worthy of further investigation.

### Conclusions and Perspectives

**Figure 8:**
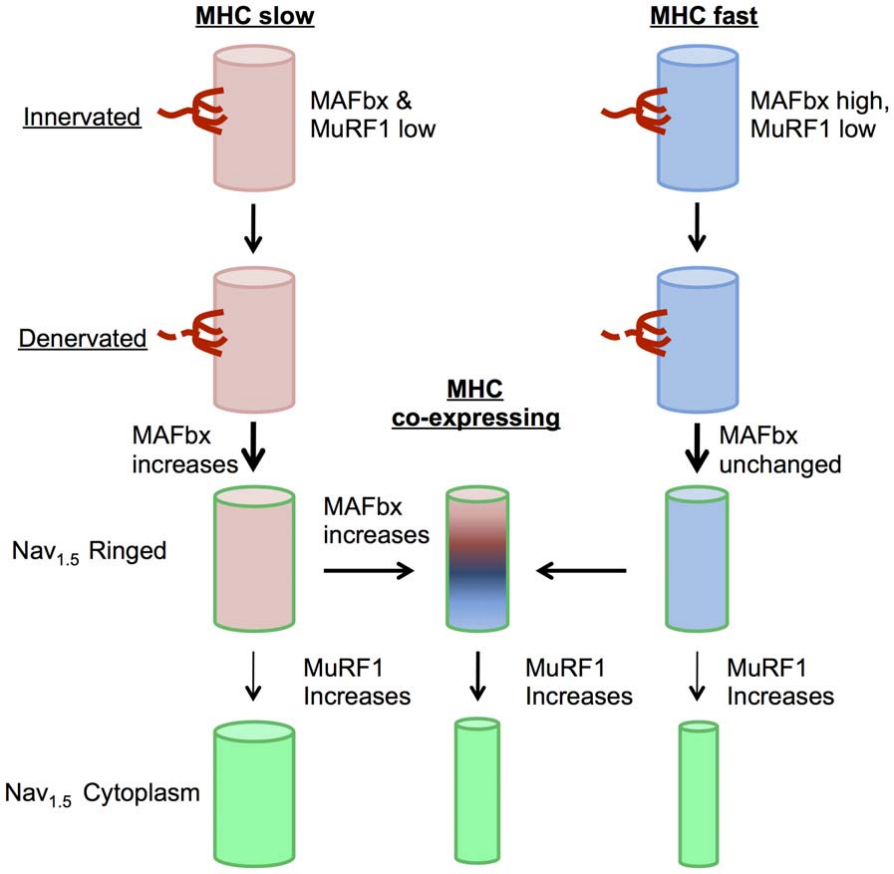
Schematic representation of atrophy in MHC slow versus MHC fast myofibers following denervation in aging muscle. Thickness of arrow indicates relative proportion of fibers taking a given path. Briefly, upon denervation, MHC slow fibers exhibit increased MAFbx expression, followed by the majority of denervated fibers beginning to co-express MHC fast, coincident with initiation of atrophy. Upon denervation of MHC fast fibers, MAFbx is unchanged but atrophy is already seen, followed by a majority of denervated fibers beginning to co-express MHC slow. MuRF1 increases with prolonged duration of denervation in all MHC categories and is associated with further atrophy in MHC fast and MHC co-expressing myofibers.

Although there are many previous studies providing evidence of denervation in aging muscle, this is the first study to quantify the degree to which denervation explains myofiber atrophy and MHC co-expression in an advanced stage of sarcopenia, suggesting that denervation is the primary factor explaining both phenomena in aging muscle (see conceptual schematic in Fig. 8). These findings have important implications for our understanding of sarcopenia, particularly at advanced stages where complications such as an increased susceptibility to falls, mobility impairment, and physical frailty are more likely to occur [55]. Specifically, our findings implicate denervation as the primary event causing the marked accumulation of severely atrophic myofibers shown previously to characterize the accelerating phase of muscle atrophy with aging [41]. Thus, accumulation of denervated myofibers may be a critical feature of sarcopenia distinguishing it from other muscle wasting conditions. As such, pharmacological strategies targeting atrophy pathways recommended for other muscle wasting conditions (e.g., [69]) may be of little effect in preventing the most significant consequences of sarcopenia. For example, since the majority of the atrophy seen in aging muscle appears to occur in denervated fibers, pharmacological interventions preventing their atrophy would place a larger burden on the remaining innervated fibers and further impair function of the aging muscle. We thus recommend renewed focus on the causes of denervation in aging muscle to facilitate development of the most effective therapeutic treatments for sarcopenia and its functional consequences.

## Materials and Methods

### Ethics Statement

All experimental procedures on animals were made with prior approval of the University of Calgary Animal Care Committee, protocol ID BI09R-11.

### Animals and Tissue Harvest

Male Fisher 344×Brown Norway F1 hybrid (F344BN) rats were obtained from the National Institute on Aging (NIA; Baltimore, MD). The animals were obtained at ages of 8–10 months (YA) and 36 months (SEN), with 6 animals per group. Upon arrival at the Biologial Sciences vivarium at the University of Calgary, all animals were housed singly in standard cages fitted with filter bonnets, and provided with food and water fed *ad libitum* (12/12 hour light/dark cycle, 21°C).

Animals were anesthetized with sodium Pentobarbital (55–65 mg×kg^−1^) and the gastrocnemius (Gas) muscle was removed, dissected free of fat and weighed. A portion through the entire midbelly was mounted on cork in optimal cutting temperature compound and frozen in liquid isopentane cooled in liquid nitrogen. Samples were stored at −80°C until sectioning.

### Spinal Cord Immunohistochemistry

The entire lumbar region of the spinal cord was dissected out of 6 YA and 5 SEN animals, and fixed in 4% formalin (Sigma) for two months. Subsequently, fixed spinal cords were cut into 1 cm fragments, processed and embedded in paraffin blocks. The tissue blocks were cut on a microtome into 50 µm-thick sections. Every tenth section was used for the stereological analysis (see below). Motoneurons were identified by immunolabelling for Choline acetyltransferase (ChAT, catalogue number AB144P, Millipore) carried out on free-floating sections. Briefly, the sections were dewaxed by overnight incubation in Histo-Clear II (National Diagnostics), rehydrated by moving them through a gradient of alcohols (100%, 100%, 90%, 70%, 50% and 30%) and rinsed in TBST. The sections then underwent heat-induced antigen retrieval in 0.1 M EDTA (pH 9.0) in a 90°C water bath for 40 min. The sections were allowed to cool to room temperature and endogenous peroxidase was blocked by incubation with 3% H_2_O_2_ in TBST for 15 min. Non-specific protein interactions were blocked by incubation with blocking solution containing 5% normal horse serum (Sigma) in TBST, with 0.1% Triton X-100 for 1 hr at room temperature. The sections were incubated overnight at +4°C in primary antibody diluted in the blocking solution at 1∶200. The following day the sections were washed in TBST and incubated with secondary antibody, anti-Goat-biotin (Vector), at 1∶200 for 2 hr. ABC complex (Vector Laboratories) was applied onto the sections for 30 min and the signal was developed with diaminobenzidine (DAB) chromogen (SigmaFast tablets, Sigma). The sections were mounted on glass slides using hard-set mounting medium (Vectashield, Vector).

### Spinal Cord Stereology

Numbers of motoneurons were obtained using stereological software – Stereo Investigator 8.0 (MBF Bioscience). Motoneurons were counted in the ventral horn of every tenth section using an optical fractionator probe to obtain unbiased counts. The optical fractionator probe permits estimation of an entire population size. The principle is based upon systematically randomized sampling of a region of interest to yield an unbiased number of cells within the volume. On average, 50±0.7 and 55±1.3 sections per animal were analyzed in YA and SEN groups, respectively. Criteria for inclusion were based on the intensity of staining (darkest cells with visible unstained nucleolus) and a soma size larger than the majority of more faintly stained cells in the same area. The entire area of Lamina IX in the ventral horn was selected in each tissue section and all of the positive cells fitting the above criteria within this area were counted. This experimental setting provided the right population size to be able to be confident with the overall population numbers estimated by the software (Coefficient of Error (Gundersen, m = 1) = 0.05). Motoneuron population size was calculated using an estimated total by number-weighted section thickness formula. The resulting motoneuron numbers were then divided by the length of the analysed spinal cold fragment and normalized to a 1 mm length of spinal cord.

### Gastrocnemius Muscle Immunolabeling

All muscle immunolabeling experiments were conducted according to methods reported previously [25]. Briefly, 10-μm thick sections were cut on a cryostat (−18°C) and mounted on lysine coated slides (Superfrost). Slides were air dried for one hour, then stored at −80°C. On the day of immunolabeling, slides were removed from the −80°C freezer and allowed to reach room temperature. The slides were first re-hydrated in phosphate buffered saline (PBS; pH 7.4) and then incubated in permeabilization solution (0.1% Triton X-100 in PBS) for 15 min. Slides were washed in three changes of PBS, incubated with blocking solution (10% goat serum, 1% bovine serum albumin in PBS; or 10% donkey serum, 1% bovine serum albumin in PBS) for 30 min, and then incubated with primary antibodies overnight at 4°C. One slide per sample was labelled with anti-dystrophin (1∶200 dilution, catalogue number D8168, Sigma), anti-MHC slow (1∶10 dilution; catalog number NCL-MHCs, Novocastra), and anti-Nav_1.5_ (1∶100 dilution, catalogue number AB5493, Millipore) antibodies. A serial section was labeled with anti-dystrophin and anti-Nav_1.5_ as above, and anti-MHC fast (1∶10 dilution, catalogue number NCL-MHCf, Novocastra). This permitted examining Nav_1.5_ labeling in relation to the expression pattern of MHC slow and MHC fast. Two other serial sections per muscle were first fixed in 4% paraformaldehyde (in PBS) after being brought to room temperature and then labeled for MHC slow and dystrophin as above, and either MAFbx (1∶100 dilution, catalogue number AP 2041, ECM Biosciences) or MuRF1 (1∶100 dilution, catalogue number , GTX 24125 Genetex). The following day, sections were rinsed three times with PBS and incubated with blocking solution for 30 min. Sections were incubated for one hour with a secondary antibody cocktail including goat anti-rabbit AlexaFluor 546 (1∶200 dilution; Invitrogen) or donkey anti-goat Alexafluor 546 (1∶200 dilution; for MuRF1), and goat anti-mouse AlexaFluor 633 (1∶200 dilution; Invitrogen). Slides were rinsed three times with PBS, rinsed with distilled water, then cover slipped using ProLong Gold. Slides were stored at 4°C until imaging. The specificity of the MAFbx and MuRF1 antibodies we used was assumed on the basis of the datasheet provided by the respective manufacturers. In this respect, the datasheet for the MAFbx antibody (AP2041, ECM Biosciences) provides a Western blot showing that this antibody identifies a single band at a molecular weight of approximately 41.5 kD in both diaphragm and gastrocnemius muscle from mouse. The datasheet for MuRF1 antibody (GTX 24125) specifies that the immunogen targets sequence DYKSSLIQDGNPM from the N terminus of the protein, but that no Western blots have been performed yet.

### Confocal Imaging and Image Analysis

Fluorescent imaging was completed on an Olympus Fluoview confocal microscope. For each muscle, two images containing ∼50 fibers each were collected from the red region of the Gas at a magnification of 200x, as done previously [70]. Serial images were collected of the same fibers for the Nav_1.5_, MHC slow, MHC fast, MAFbx, and MuRF1 labels. Images were imported from the Fluoview software into Image J (version 1.44, NIH) for analysis. The cross-sectional area of fibers in each image was determined, using an internal reference frame within the image to prevent bias, as done previously [71]. Fibers were subsequently categorized as pure MHC slow, pure MHC fast or MHC co-expressing (demonstrating both MHC slow and MHC fast labelling) fibers using images from the serial section. The Nav_1.5_ status was classified in these same fibers. In serial sections, the fluorescence intensity of MAFbx and MuRF1 were quantified using Image-J in the same fibers in which Nav_1.5_ status and MHC labeling pattern was determined.

### Statistics

Values are presented as means±SE. For all statistical comparisons of YA vs SEN, a student's T-test was used. Comparisons between MHC classifications were performed using one-way ANOVAs, with a Holm-Sidak post-hoc test. When ANOVA's failed due to unequal variance, a Kruskal-Wallis one-way ANOVA on ranks was completed, with a Tukey post-hoc test. For comparisons of MAFbx and MuRF1 expression differences between Nav_1.5_ positive and Nav_1.5_ negative were done by two-way ANOVA (Nav_1.5_ status x MHC expression), and a Holm-Sidak post-hoc test. For all statistical analyses, α = 0.05.

## Supporting Information

Figure S1 **Negative controls for Nav_1.5_ experiments.** A SEN GAS section was incubated without the primary antibody, to test for non-specific binding of the secondary antibodies. Nav_1.5_ channel (Panel A) and MHCs/dystrophin channel (Panel B) images demonstrate the lack of non-specific labelling. Scale bar is 50 µm.
(TIFF)

Figure S2 **Positive controls for Nav_1.5_ experiments.** A heart section was tested for non-specific secondary antibody binding. Nav_1.5_ channel (A) and dystrophin channel (B) images demonstrate the lack of non-specific labelling. A serial section was incubated with primary antibodies and confirms the presence of Nav_1.5_ in cardiomyocytes (C); individual cardiomyocytes are identifiable by dystrophin labelling (D). Scale bar is 50 µm.
(TIFF)

Figure S3 A: Fiber size distribution according to the pattern of Nav_1.5_ expression in the red region of gastrocnemius muscle of senescent animals. B: Fiber size distribution of Nav1.5 positive fibers (ringed plus cytoplasm combined) according to the MHC labeling category in the red region of gastrocnemius muscle of senescent animals.
(TIFF)

Figure S4 **Cross-sections incubated without primary antibody (negative control) for MAFbx (A) and MuRF1 (B), demonstrating no non-specific labeling.**
(TIFF)
