# Supplementary figures and images for: Denervation Causes Fiber Atrophy and Myosin Heavy Chain Co-Expression in Senescent Skeletal Muscle
Source: PLoS One. 2012 Jan 3;7(1):e29082. doi: 10.1371/journal.pone.0029082 (PMC3250397; doi:10.1371/journal.pone.0029082)

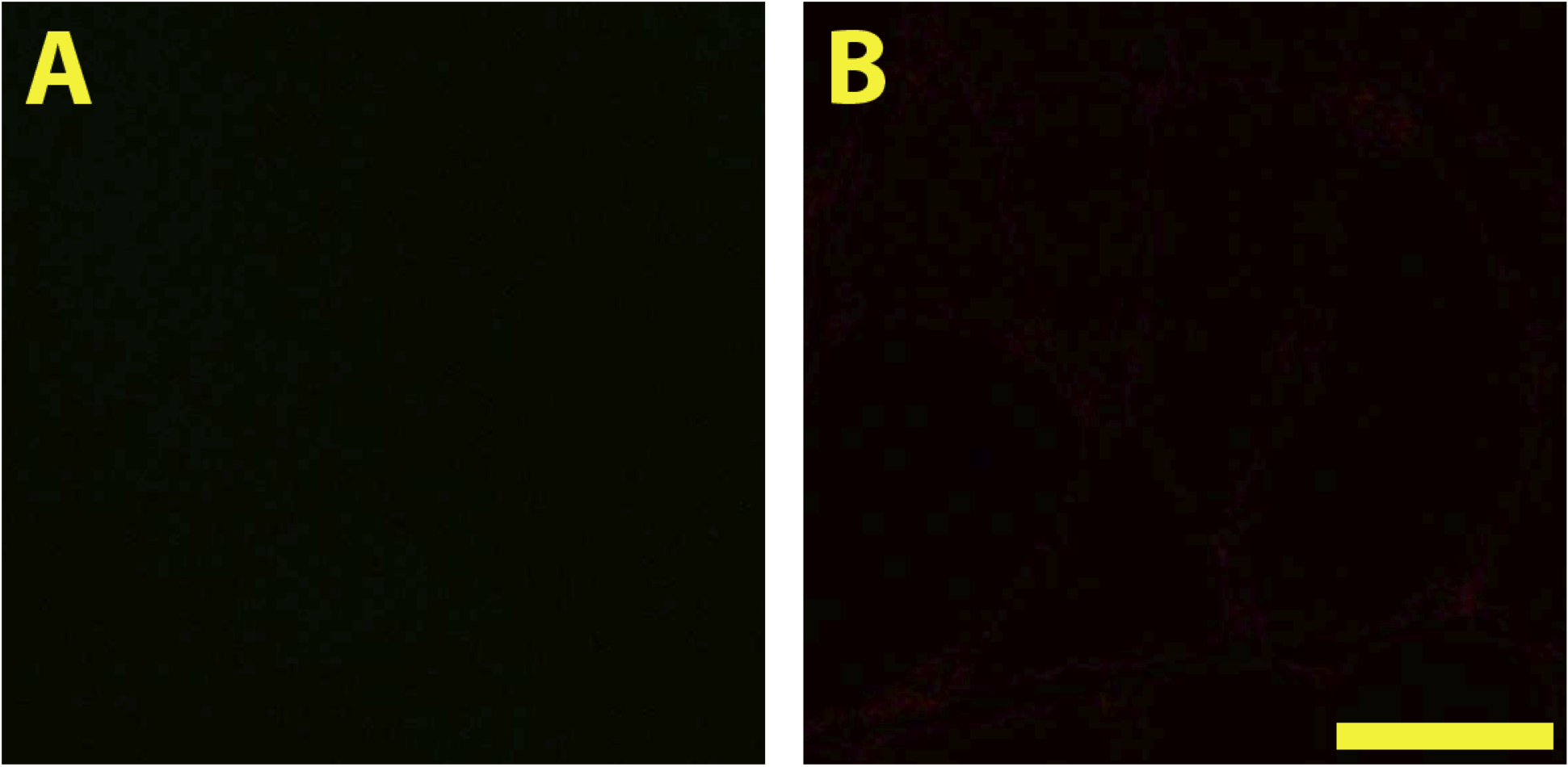

Supplement: Figure S1 — Negative controls for Nav1.5 experiments. A SEN GAS section was incubated without the primary antibody, to test for non-specific binding of the secondary antibodies. Nav1.5 channel (Panel A) and MHCs/dystrophin channel (Panel B) images demonstrate the lack of non-specific labelling. Scale bar is 50 µm. (TIFF) [file pone.0029082.s001.tif]

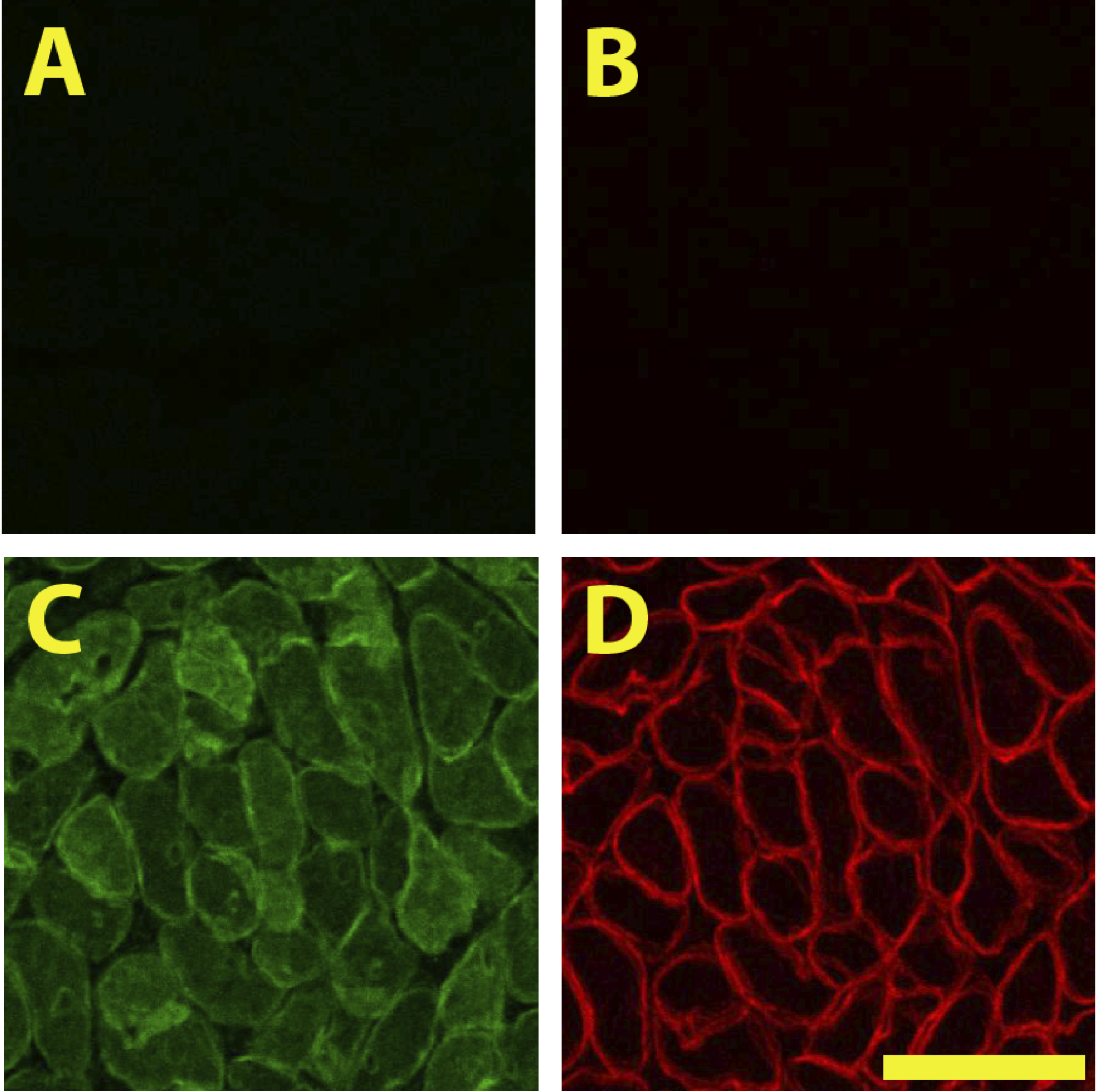

Supplement: Figure S2 — Positive controls for Nav1.5 experiments. A heart section was tested for non-specific secondary antibody binding. Nav1.5 channel (A) and dystrophin channel (B) images demonstrate the lack of non-specific labelling. A serial section was incubated with primary antibodies and confirms the presence of Nav1.5 in cardiomyocytes (C); individual cardiomyocytes are identifiable by dystrophin labelling (D). Scale bar is 50 µm. (TIFF) [file pone.0029082.s002.tif]

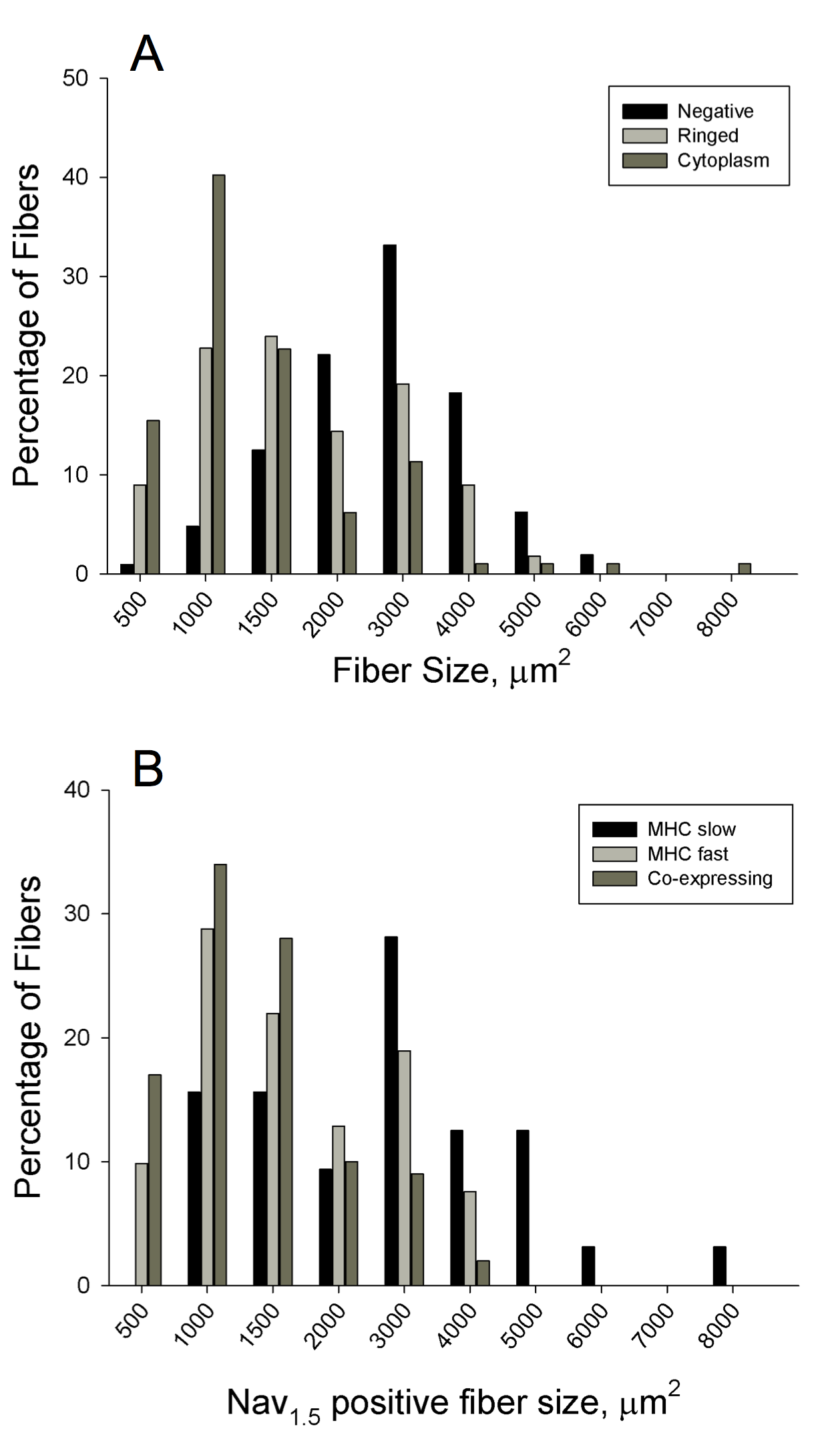

Supplement: Figure S3 — A: Fiber size distribution according to the pattern of Nav1.5 expression in the red region of gastrocnemius muscle of senescent animals. B: Fiber size distribution of Nav1.5 positive fibers (ringed plus cytoplasm combined) according to the MHC labeling category in the red region of gastrocnemius muscle of senescent animals. (TIFF) [file pone.0029082.s003.tif]

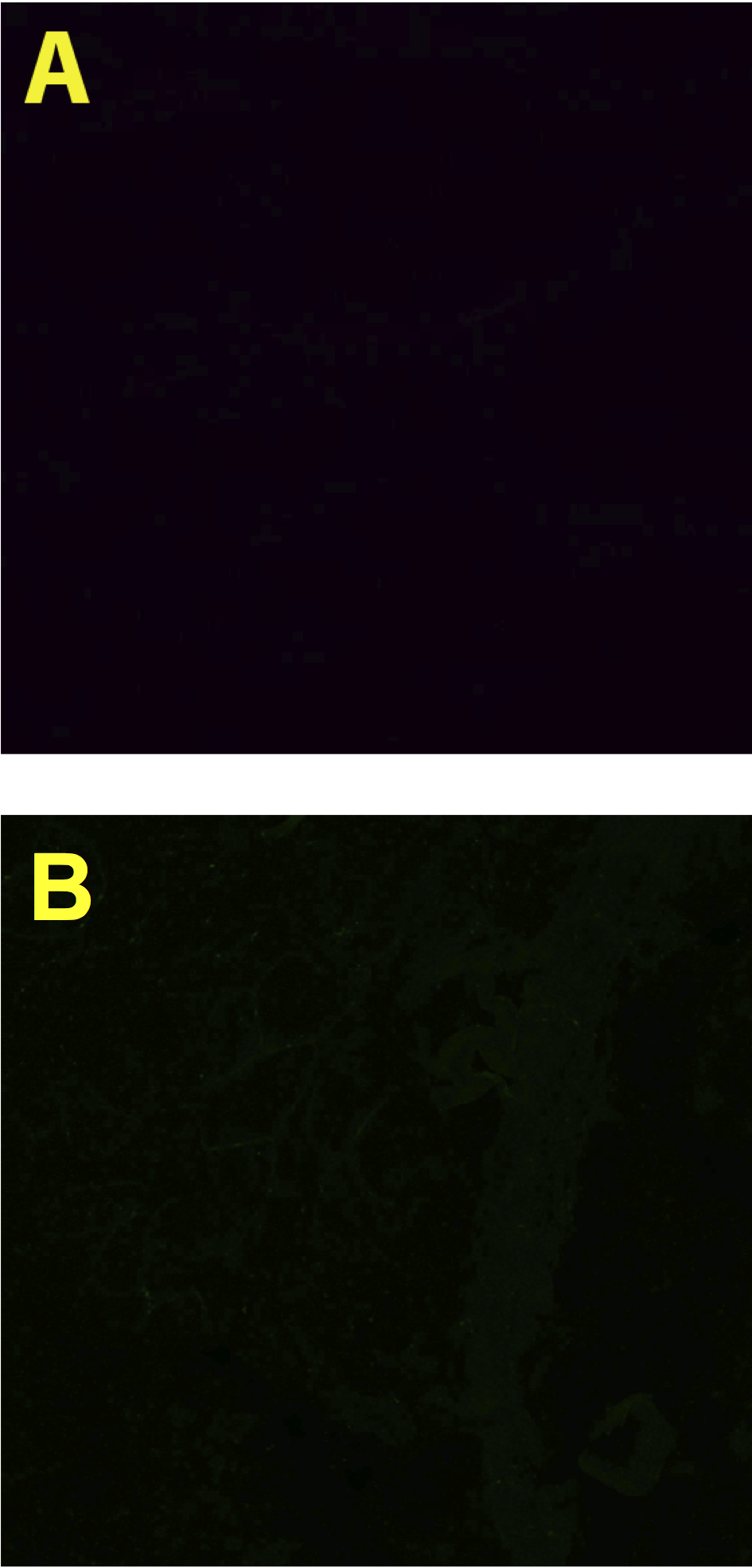

Supplement: Figure S4 — Cross-sections incubated without primary antibody (negative control) for MAFbx (A) and MuRF1 (B), demonstrating no non-specific labeling. (TIFF) [file pone.0029082.s004.tif]
